# ‘Counselling is not just providing information’: perceptions of caregivers and stakeholders on the design of nutrition and health counselling interventions for families with young children in rural Kenya

**DOI:** 10.1186/s12913-024-10872-w

**Published:** 2024-05-07

**Authors:** Grace Wothaya Kihagi, Lea-Sophie Hansen, Erick Agure, Erick M.O. Muok, Isabel Mank, Ina Danquah, Raissa Sorgho

**Affiliations:** 1 Heidelberg Institute for Global Health (HIGH), Medical Faculty and University Hospital, University of Heidelberg, Heidelberg, Germany; 2 Kenya Medical Research Institute (KEMRI), Kisumu, Kenya; 3 German Institute for Development Evaluation (DEval), Bonn, Germany; 4 Hertz-Chair Innovation for Planetary Health and Center for Development Research (ZEF), University of Bonn, Bonn, Germany; 5 Centre for Wellness and Nutrition, Public Health Institute, Sacramento, CA USA

**Keywords:** Nutrition and Health Counselling, Rural Kenya, Child Nutrition, Behaviour Change Communication, Sub-Saharan Africa.

## Abstract

**Background:**

Globally, a fifth of the children continue to face chronic undernutrition with a majority of them situated in the Low- and Middle-Income Countries (LMIC). The rising numbers are attributed to aggravating factors like limited nutrition knowledge, poor feeding practices, seasonal food insecurity, and diseases. Interventions targeting behaviour change may reduce the devastating nutrition situation of children in the LMICs.

**Objective:**

For the co-design of a Behaviour Change Communication (BCC) intervention for young children in rural Kenya, we aimed to identify the experiences, barriers, facilitators, and preferences of caregivers and stakeholders regarding nutrition and health counselling.

**Design:**

We employed a qualitative study design and used a semi-structured interview guide. The in-depth interviews were recorded, transcribed, and analysed using content analysis, facilitated by the software NVivo.

**Setting:**

Health and Demographic Surveillance System (HDSS) area in Siaya County, rural Kenya.

**Participants:**

We interviewed 30 caregivers of children between 6 and 23 months of age and 29 local stakeholders with experience in implementing nutrition projects in Kenya.

**Results:**

Nutrition and health counselling (NHC) was usually conducted in hospital settings with groups of mothers. Barriers to counselling were long queues and delays, long distances and high travel costs, the inapplicability of the counselling content, lack of spousal support, and a high domestic workload. Facilitators included the trust of caregivers in Community Health Volunteers (CHVs) and counselling services offered free of charge. Preferences comprised (1) delivering of counselling by CHVs, (2) offering individual and group counselling, (3) targeting male and female caregivers.

**Conclusion:**

There is a disconnect between the caregivers’ preferences and the services currently offered. Among these families, a successful BCC strategy that employs nutrition and health counselling should apply a community-based communication channel through trusted CHVs, addressing male and female caregivers, and comprising group and individual sessions.

**Supplementary Information:**

The online version contains supplementary material available at 10.1186/s12913-024-10872-w.

## Introduction

Childhood undernutrition continues to be a global challenge and poses a threat to achieving the Sustainable Development Goals (SDGs). There has been a decline in childhood chronic undernutrition (stunting) from 203 million in 2000 to 149 million in 2020. Still, every fifth child < 5 years of age has chronic undernutrition. The majority of these children (38%) are living in sub-Saharan Africa [1]. In Kenya, the proportion of children suffering from stunting is 18%, while 5% of children have acute undernutrition [2]. Poor child-feeding practices have been associated with child undernutrition, particularly in Sub-Saharan Africa [3, 4]. In rural Kenya, caregivers among small-scale farmers assign little value to local and traditional foods and show limited nutrition knowledge. This has resulted in the consumption of energy-dense but micronutrient-scarce foods [5, 6]. In addition, aggravating factors in Siaya County, Kenya, like HIV infections (Prevalence: 21%), malaria infections (Prevalence: 54%) and seasonal food insecurity predispose under-fives to poor health and undernutrition [7, 8].

Behaviour change communication (BCC) interventions constitute a promising strategy to improve the health and nutrition of young children by empowering caregivers with the skills to provide healthy feeding for their children [5, 9]. BCC is defined as an interactive process with communities to develop tailored messages and approaches using a variety of communication channels. BCC aims at developing and maintaining positive behaviours that promote and sustain individual, community, and societal change [10]. Nutrition and health counselling (NHC) forms one of these BCC strategies: A trained counsellor educationally conducts counselling sessions to facilitate changes in food choices, hygiene, utilization, feeding, and care-seeking. NHC is a supportive process between a counsellor and a client to set priorities, establish goals, and create individualized action plans that acknowledge and foster responsibility for self-care [11]. A trained counsellor provides training on maternal, infant, and young child nutrition (MIYCN) to improve feeding practices and health behaviours. In Kenya, counselling on MIYCN mainly takes place at mother and child clinics during antenatal care visits, yet only 4 out of 10 women access the ANC services [12]. Evidence shows positive associations between the uptake of MIYCN counselling, improved feeding practices, and the reduction of stunting [13–15].

The successful adoption of BCC requires that clients recognize the value of NHC, and feel motivated to adopt counselled practices [11]. Studies on the perception of such counselling among inpatients show an association between adherence to dietary recommendations and improved dietary habits [16]. Despite this well-acknowledged need to understand perceptions and preferences regarding BCC with NHC, we lack contextualized information from caregivers living in rural Kenya and stakeholders with nutrition expertise.

Therefore, this qualitative study aimed to explore the perceptions and preferences of caregivers living in rural Siaya and local stakeholders to determine how best to design a nutrition and health counselling BCC intervention. The specific objectives were (1) to describe caregivers’ and stakeholders’ experiences of NHC, (2) to identify and describe barriers to and facilitators of such counselling, and (3) to determine their respective preferences.

## Methods

To ensure the quality of reporting our findings, we applied the Consolidated Criteria for Reporting Qualitative Research, (COREQ) guidelines in the entirety of this qualitative research process [17]. The guidelines were developed to help researchers focus on the relevant areas, which must be reported in qualitative research [17, 18]. COREQ guidelines have a checklist containing 32 specific criteria for the three domains namely, the research team and reflexivity, the study design, and data analysis and reporting of findings. In the Supplementary material (01), attached, we have documented how each section was covered.

### Study setting

**Fig. 1:**
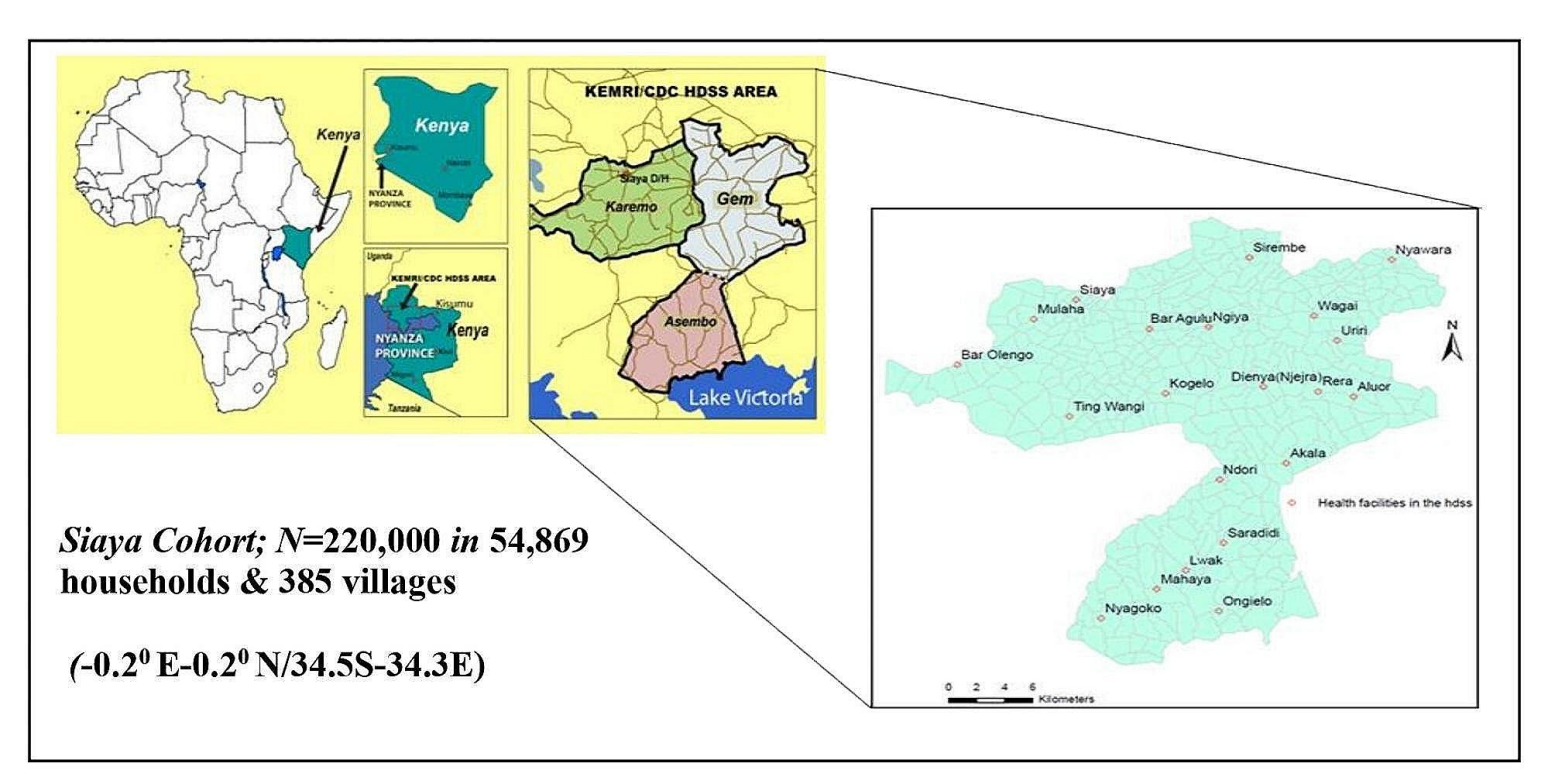
Map of the Siaya health and demographic surveillance system (HDSS) area, adapted from KEMRI HDSS, 2019

This qualitative study was conducted within the Siaya Health and Demographic Surveillance System (HDSS) area in South-Western Kenya, located in the North-East of Lake Victoria with approximately 1 million inhabitants [2]. Study participants were recruited from the villages of Gem, Karemo, and Asembo of Siaya County (Fig. 1), where people mainly speak the Dholuo language. The primary occupation of most inhabitants is smallholder subsistence farming, complemented by fishing activities. Additionally, subsistence farming contributes to 70–90% of livelihood in this area, and starchy staples and legumes make up a typical diet according to the Living Standards Measurement Study (LSMS) of the World Bank.

### Population, sampling, and recruitment

Purposive sampling was applied to the Siaya HDSS population for the recruitment of 10 caregivers of under-fives from each of the three regions within Siaya HDSS; Gem, Karemo, and Asembo, totalling to 30 caregivers. The eligibility criteria for caregivers were (1) being the primary caregiver of a child aged 6–23 months, (2) having lived in rural Siaya for at least two years before the study started, and (3) being conversant in Dholuo, Swahili, or English languages. We used a combination of purposive and snowball sampling to identify and recruit the stakeholders. First, we mapped out stakeholders involved in nutrition and home gardening projects within Siaya from prior scoping review conducted by two researchers (RS, LH) [19]. We identified 116 potential stakeholders working within 2 years in Non-Governmental Organizations (NGOs), Community-Based Organizations (CBOs), or Government ministries. We sent initial and follow-up emails to the eligible stakeholders and began the recruitment process while encouraging them to share the names of other professionals fitting the eligibility criteria. Sampling and recruitment continued until data saturation and redundancy were reached [20, 21] A total of 29 stakeholders were recruited. Of the participants contacted, none declined to participate.

### Data collection

Between September and October 2020, three well-trained researchers, two females and one male (GWK, LH, EA) conducted interviews once; they had no previous contact with the study participants. For the interviews with caregivers, we used a semi-structured interview guide (Supplementary material 02). Two linguistically matched researchers (GWK, EA) contacted the caregivers at home and sought consent to conduct the in-person interviews. One of the researchers conducted the interview, and the other one took notes, i.e., documenting the process, key points, and observations. All interviews with caregivers were audio-recorded at the participants’ homes. The interviews lasted for an average of 37 min.

The interviews with stakeholders were conducted in the two official languages in Kenya: English and Swahili, by two researchers (GWK & LH), based on individual participant’s preference. The researchers used a semi-structured interview guide (Supplementary material 03), which was piloted and tested before data collection. It contained broad topics drawn from the study objectives, with key questions to guide the discussions [22]. We started to conduct in-person interviews in the stakeholders’ offices but had to transition to a digital video conference tool because the COVID-19 pandemic ruled out in-person meetings. Of the 29 interviews conducted, only two were in-person, the rest were conducted on digital platform. The interviews lasted between 60 and 100 min, were audio-recorded, and saved in a password-protected cloud system. The notetaker documented all additional material for cross-referencing and interview analysis. After each interview, the interviewer and the notetaker conducted a verbal debriefing of the interview and completed a debriefing form. We later used interview notes and debriefing forms as source material for triangulation [23].

Throughout the data collection period, the researchers (GK, LH, EA) held bi-weekly debrief meetings with the research team to check on the progress of the interviews, the emerging points of interest in the data, and monitoring data saturation [20]. Finally, two translators transcribed the interviews verbatim [24] and translated them from Dholuo or Swahili to English before being uploaded to the software NVivo, version 12 for analysis.

### Data analysis

The interview transcripts, field minutes, and observation notes were read to inductively identify points of interest for the directed content analysis [25]. Two researchers (GK, LH) conducted two rounds of coding to identify any new codes to expand the coding frame [26, 27]. The first round was done independently on half of all the caregivers’ and stakeholders’ transcripts. NVivo was used to organize, store, and visualize the data [28]. Next, we compared our preliminary codebooks, defined our codes, explained them to each other, and discussed points of concurrence and divergence. Afterward, we built a combined coding frame that served as the basis for the second coding round. Upon agreement on the coding frame, which remained open to additions and flexible to change, we conducted a second round of coding for all the transcripts. Lastly, during the second round of coding, categories were developed and then linked with the overarching concepts [29]. To improve the validity and the quality of our findings, we triangulated them with data from observation notes and debriefing forms [23].

### Dissemination and validation of findings

To enhance the trustworthiness of our findings, we held a virtual feedback and dissemination meeting with the interviewed stakeholders in March 2021 [30–32]. Prior to the meeting, we sought consent from stakeholders to participate in a group meeting. Participants were given the option to remain anonymous in the group meeting or have an individual debriefing session. A total of 11 participants consented and participated. Dissemination of findings for caregivers was not possible due to COVID-19-related travel restrictions.

## Results

We present participants’ responses to our three above-mentioned research objectives using representative quotes [33]. Quotes were labelled with the following information: participant group (Caregiver-CG, Stakeholder-SH), study-specific ID number (01 to 30), organization category and participant role or relationship to the child, respectively, and age of the participant.

### Participants’ characteristics

Out of the 30 interviewed caregivers, 27 were mothers and 3 were grandmothers (Table 1) Regardless of their relationship to the index child (mothers or grandmothers), the two sets of caregivers in our study were defined as the primary caregivers, who took full custody and care of the children. Their age ranged from 22 to 68 years, and mean duration of schooling was 8.7 years (range: 0–14 years).

**Table 1:** Socio-demographic characteristics of the study participants

Characteristics	Caregivers		Stakeholders
N	30		29
Age (years)	31.5 (22–68)		43.0 (26–67)
Female sex	100 (30)		41.4 (12)
Experience with nutrition counselling			
With experience	80.0 (24)		75.9 (22)
Without experience	20.0 (6)		24.1 (7)
Group			
Mother	90.0 (27)	Governmental ministries	13.8 (4)
Grandmother	10.0 (3)	Research & academia	13.8 (4)
		Non-governmental organizations	72.4 (21)

Data are presented as means and ranges for continuous variables and as proportions in % (n).

The majority of caregivers (24/30) had already received some sort of nutrition education or counselling. Of the 29 interviewed stakeholders, the majority (21/29) worked with NGOs, including national, local, and community-based. Four of the stakeholders worked for government ministries, while the remaining four were employed at research institutions. Almost half (12/29) of the stakeholders were women. They were aged between 26 and 67 years, and 22 of them had experience with nutrition education or counselling projects implemented in Kenya.

## Experiences of caregivers and stakeholders

### Reasons for seeking and initiating nutrition and health counselling

Of the caregivers with past nutrition counselling experience, three main reasons were reported for seeking nutrition counselling:

1) Knowledge gain: Caregivers wanted to learn from experts about child feeding, *“I went so that I can receive this training on food nutrition because I might have given birth when I didn’t know anything, and I don’t know what is going on. That can make me make a lot of mistakes, for example, giving the baby food too early…” (CG_019; Mother, 25).*

2) Address feeding and health problems: Caregivers intended to address the poor feeding habits of their infants to improve their health, *“I thought counselling could help me because sometimes she [my child] was not eating and I couldn’t do anything. The child was sick and was not eating, so I went to hospital where I was told to look for a place where I can get trained on how she should eat so that she can have good health…”* (*CG_017; Grandmother, 51*).

3) Externally initiated: The health facilities have created an opportunity for nutrition and health counselling by offering it as part of an integrated service to mothers attending antenatal or postnatal care, “*I went [to the nutrition counselling] because I was also taking the child to clinic, and every time we took them to clinic, there were days for training, so I used to sit for those trainings”(CG_020; Mother 26*). In some instances, the health providers also deliberately requested that the caregiver attends counselling, *“Nurses told us that there’s counselling so we should not leave” (CG_011; Mother, 40)*.

From the perspectives of the stakeholders, there were 5 reasons to conduct nutrition and health counselling for caregivers of young children:

1) Improve nutrition and health status of the children: The stakeholders initiated projects with nutrition components, first, as a strategy to improve the deteriorating health and nutrition status of children, and second, as a solution to Human Immunodeficiency Virus (HIV)-related malnutrition and food insecurity, “*we had to start it [nutrition project] because when we use to make the home visits, there were a lot of children who were HIV infected, they were not able to get good nutrition….” (SH_023;Program officer/ NGO, 34).*

2) Empower the community: Stakeholders stated they need to empower the community through capacity building, “*The main reason is just to improve the health of the community. In fact, if they know what they should eat, and what they should not, and the balance of all those foods, different categories of foods, it will improve the nutritional status of the community.” (SH_015;Home-economics officer/ GOK, 43).*

3) Target critical periods of child growth and development: Stakeholders initiated nutrition projects to optimize child health during the first 1000 days of life, the period between pregnancy until 2 years of age, “… *The process starts while the child is in the womb. So, when you start nutritionally counselling the mother, then mother nutritionally implement those advice for the benefit of the child” (SH_009; Nutritionist/GOK, 47).*

4) Target poor feeding practices: Stakeholders intended to address poor child feeding practices through BCC for the caregivers, *“…a lot of children in the slums are fairly wasted because there is typical competition for good nutrition between the adults and the children who should eat…” (SH_012; Program officer/NGO, 45).*

5) Nutrition and health counselling was part of an integrated project: A few stakeholders explained that nutrition was part of an integrated approach in their projects, that aimed to improve household resilience and empower women, e.g., by home gardening, “*…the nutrition is also part of the home gardening. So, it’s not really a project on nutrition, but it’s like an additional point to home gardening…” (SH_017; Program officer/CBO, 41).*

### Current mode of delivery

Both participant groups reported that nutrition and health counselling was mostly offered in hospitals (Table 2). The target audience for these sessions was mothers. Fathers rarely received counselling on child nutrition, because they seldomly accompany mothers to the hospital. However, some caregivers highlighted that with a change in counselling setting, from a hospital to the community, more men attended the sessions. NHC sessions took place monthly and lasted between 1 and 2 h. Caregivers and stakeholders highlighted that health personnel, nurses, physicians, nutrition officers, and CHVs have conducted counselling sessions.

**Table 2:** Mode of delivery for nutrition and health counselling in rural Siaya

NHC components	Codes from transcripts	Caregivers’ & stakeholders’ quotes
Setting	Health facilities setting	*“We used to be trained when we go to the clinic…” (CG_009; Mother, 32).* *“… mostly nutritional counselling is offered in the maternal and child health clinic…” (SH_013; Director/GOK, 41)*
	Community setting	*“We had to work hand in hand with the ministry of health in ensuring that they were able to train our community home volunteers, who normally visit the households” (SH_023; Program officer/NGO, 34)*
Target audience	Only mothers Only women	*“I never saw men there.” (CG_011; Mother, 40)* *“… one of the major strategies we used was actually also going to the hospitals where the mothers in the morning when they come for what we call antenatal clinics, they usually have those classes’’ (SH_004;Nutritionist/ Academia, 43)*
	Both mothers and spouses counselled	*“We don’t usually go with him [father of child]. I cannot lie, we have never gone with him” (CG_016, Mother, Unknown age)* *“We went with him for the first time, and I went alone for the rest” (CG_020; Mother, 26)* *“When we started, it was only women. But when they [CHVs] started coming to meet us in the village, they had to be open and talk to our men also” (CG_004, Mother, 22)*
Duration of counselling session	30–60 min 60–90 min	*“Just half an hour to one hour”. (CG_024; Mother, 38)* *“It would take even an hour” (CG_004, Mother, 22)*
	> 90 min	*“… I have done one for three hours because there were a lot of issues that needed to be discussed so I think the longest have done is 3 h” (SH_009;Nutritionist/ GOK/NGO, 53)*
Frequency	once a month,	*“… those sisters [nurses] at Lwak hospital trained us. We meet once a month” (CG_004; Mother, 22)* “…. *when we were doing the nine months follow-up studies, it would be every month, every month they are coming for follow up, we would offer some nutritional guidance, some nutritional information” (SH_014; Nutritionist/ Academia, 39)*
	more than once a month	“…*each person is supposed to get up to two sessions per month” (SH_005; Nutritionist/NGO, 45)*
Counsellor	CHVs	*“When I take the child to the clinic to get the injections, there are usually Community Health Workers who are assigned; they usually train for a short time” (CG_029; Mother, 31)*
	Health personnel: Nurses, Nutrition officers	*“We are usually taught by the doctor when we take children to the clinic” (CG_020; Mother, 26)*

### Caregivers’ likes and dislikes

**Fig. 2:**
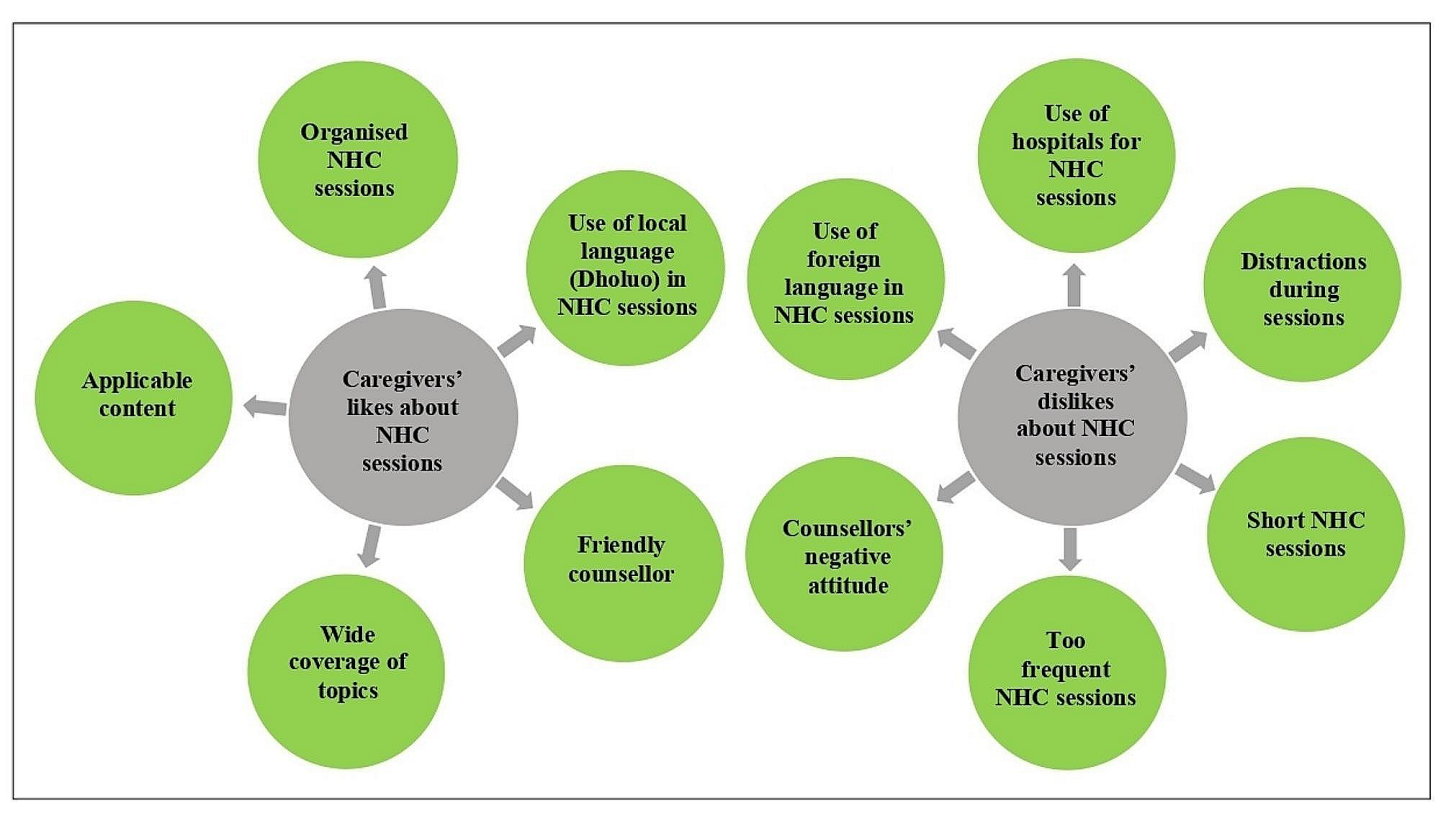
Summary of caregivers’ likes and dislikes about nutrition and health counselling

Participants shared a range of experiences from past NHC and raised a few concerns. Caregivers liked how the past sessions were organized, “*It was clearly done” (CG_006; Mother, 28).* They were happy with the content delivered and found it applicable and useful, “*It was done well. They also said that you should look for food that you can easily get… Provided you have taken balanced diet” (CG_ 001; Mother, 30).* Similar sentiments were shared by another caregiver who talked about wide coverage of topics, *“They taught us about nutrition, hygiene, how to take care of ourselves. So, all that was good for me” (CG_011; Mother, 40)*, (Fig. 2).

Concerning their experiences with the counsellors, the caregivers classified the sessions as either desirable or undesirable based on their encounters. Those who interacted with friendly counsellors enjoyed their counselling sessions, “*I used to like the one [counsellor] who used to teach because he is someone who when teaching, teaches like he is joking. He liked jokes. He used to make us happy.…” (CG_002; Mother 30).* On the contrary, those who encountered unfriendly, judgemental, boastful, or un-accommodating counsellors, classified their past counselling experiences as undesirable, *“When they [the counsellors] tried telling me that when I am unable to feed the child, there are departments that can look at your child’s well-being. That one did not please me” (CG_016; Mother, Unknown age).* Another participant recounted, *“The person taking charge [of the group counselling sessions] wants to show off to you, that can make you feel like leaving.” ****(****CG_002; Mother, 28).* Similarly, the use of local language (Dholuo language) by the counsellors during the sessions enhanced the caregivers’ desirability. They preferred sessions conducted in their local language.

Caregivers were unhappy with the hospital as the setting for nutrition and health counselling; they found the session timing inappropriate, and complained about the transportation costs to and from the health facility, *“…*we *were required to go every month, so the challenge I had was the fare [transportation cost], (CG_025, Mother, 36).* Caregivers disliked short counselling sessions, as they limited the question-and-answer part, *“Let me say that the time for asking questions was short” (CG-020, Mother 26).* Some participants of group counselling found the dynamics and side chatter as distracting, “*When you’re in a group, you hear people gossiping…could be complaining about something yet you’re trying to listen to what is being said [by the counsellor], and you end up not concentrating” (CG_006; Mother, 28).*

### Perceived effects of nutrition and health counselling

**Fig. 3:**
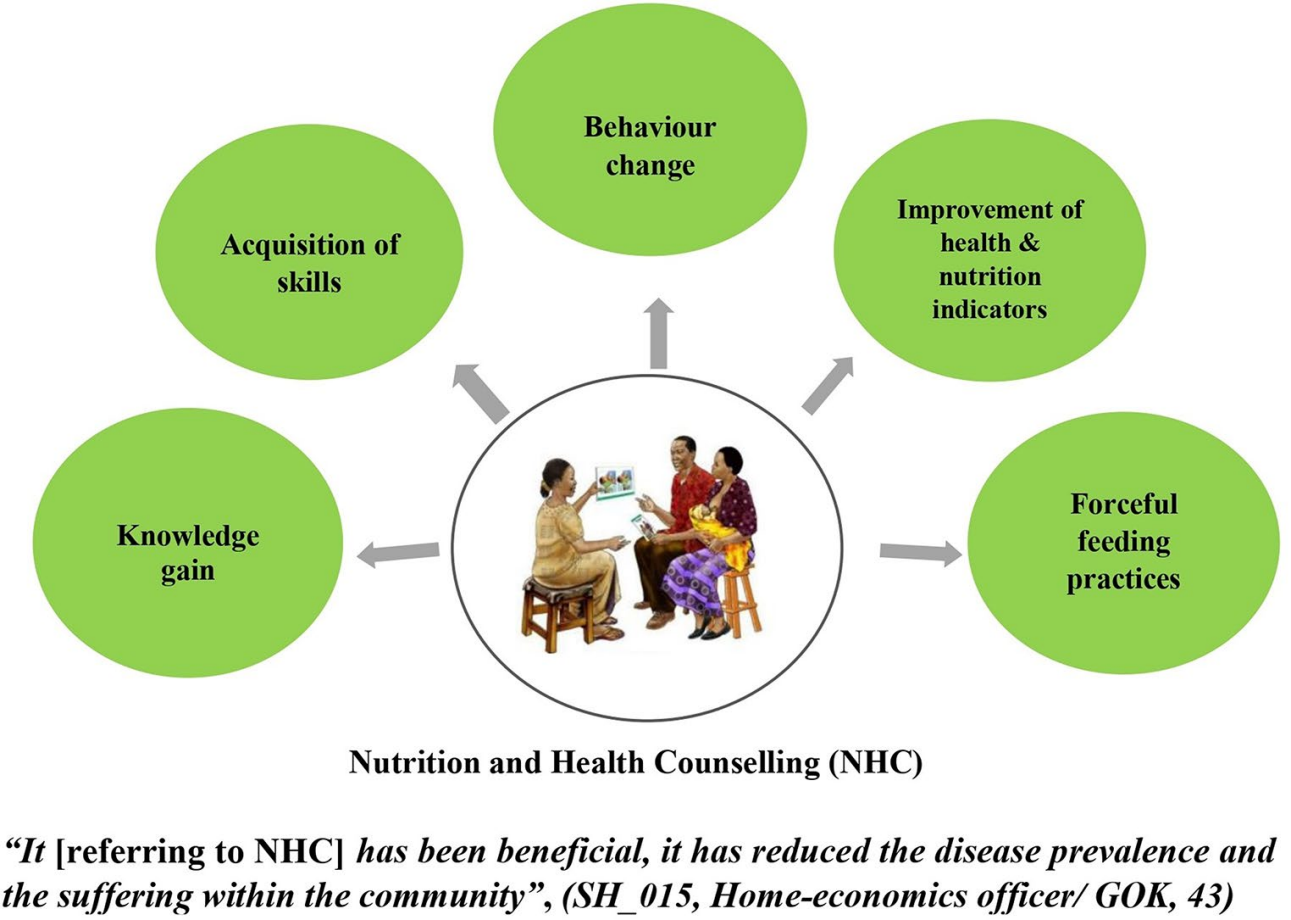
Summary of perceived effects of nutrition and health counselling

We identified five themes among caregivers and stakeholders concerning the perceived effects of nutrition and health counselling (Fig. 3).

1) Knowledge gain: Caregivers reported learning new concepts about feeding and nutrition in general, “*I will continue with them because you gave me directions, trained me on the things I didn’t know, making me enlightened, so they are things that I will continue practicing and even teaching my fellow [mothers] on how to go about them” (CG_028; Mother, 22*). This was echoed by the stakeholders, “*There’s certainly improved knowledge, if you do a pre-and post-assessment you would find that they have much more knowledge about what the practices should be…” ****(****SH_019; Nutritionist/ NGO, 45).*

2) Acquisition of skills: Caregivers mentioned that they acquired new skills in the preparation of nutritious meals, which they found applicable to their daily life, “*The benefits I can get later is that I’m taught how to cook the food and the types of foods to eat and how to change your diet” (CG_008; Mother, 24)*. This was exemplified by one stakeholder: “*Some have also come up with their own initiative and they are using the available resources to provide a balanced diet… the caregivers came up with something they used to call ‘thirty-thirty strategy’…, which was meant to feed their child for a whole day… 10 shillings is for buying a protein, 10 shillings is for buying a fruit or vegetable, and 10 shillings is for buying a carbohydrate” (SH_005; Nutritionist/NGO, 45).*

3) Behaviour change: Caregivers and stakeholders witnessed positive changes in consumption patterns and improved dietary diversity of children and the whole family, “*I have changed even on the vegetables that we should eat daily.” (CG_002; Mother, 30)*; *“After the training, I used to give the child fruits after food every day.” (CG_020; Mother, 26).* The improved dietary diversity among households was confirmed by stakeholders, *“we did see significant improvements in dietary diversity, consumption of iron-rich foods, reduction in morbidities…., so we think that most of the effect was likely due to the monthly trainings”, (SH_25,26,27; Program officers/ International Organization, 27,28,33).*

4) Improvement of health and nutrition indicators: Both caregivers and stakeholders alluded that after the counselling and application of the skills learned, they saw changes in the health and nutrition of their children, “*I could see that porridge did not give him enough satisfaction, so when he also took fruits, I could see that he had good health” (CG_018; Mother, 28). “…we see improved outcomes in terms of feeding practices, in terms of the health and nutrition status of the counselee from nutrition counselling” (SH_008; Nutritionist/GOK/NGO, 53).*

5) Forceful feeding practices: One caregiver reported having adopted an undesirable feeding habit, ‘forceful’ feeding, “…*the child was not eating properly, and I was told that there are certain foods that she should be eating so I gave her….… There are some foods which I was told ‘to force her’ to eat even if she didn’t want to” (CG_017; Grandmother, 51).* The stakeholders also highlighted forceful feeding as a common problem in Siaya. They hypothesized that its introduction was likely a result of a counsellor’s poor communication skills.

### Barriers to and facilitators of participating in nutrition and health counselling

We outline the obstacles faced by caregivers while seeking NHC (Table 3). These services were inaccessible, according to some caregivers. They also highlighted long distances to the health facilities, long queues, and delays. The inability to afford the prescribed diet and transport to the facility were among the barriers. Importantly, a lack of spousal support and the women’s workload were frequently mentioned barriers. Caregivers reported having been too occupied with childcare and household tasks to attend counselling sessions. Lastly, they were unable to attend NC when they were sick or ill.

**Table 3:** Caregivers’ barriers to nutrition and health counselling

Reported barriers	Barrier categories	Participants quotes
Time consumption	Long-distance, long queues & lengthy session & delays	*“Lwak (*Name of a hospital*) is sometimes far, sometimes you are aware that there should be a counselling session there, but you do not have means by which to reach there. That makes it hard.” (CG_001; Mother, 30)* *“From here to Siaya* [another hospital] *is far because the clinics we have around have no counselling…. There* [meaning at the hospital] *they bring people together and are counselled. For a pregnant woman, it’s tiresome because sometimes you even go on foot. So those are the challenges, that’s why sometimes we don’t even go for clinics the right way” (CG_011; Mother, 40)*
Inaccessibility	High transportation cost Lack of spousal support for participation in counselling	*“Most of the time, I get treatment at Siaya Hospital and so I must get transport” (CG_004, Mother, 22)* *“…sometimes you are called today that you are needed tomorrow, and you don’t have the fare, it is difficult for you” (CG_ 025; Mother, 36)* “*The only challenge I would have is that sometimes my husband might not allow me to go because he knows that when I go, I might not come back*” *(CG_015; Mother, 25)* *“Only if my spouse accepts. At times he can be difficult, only if he accepts. For I would be willing but only if he accepts…” (CG_012; Mother, 33)*
Inapplicability of counselling	Inaccessibility of the prescribed foods Counselling not perceived as beneficial	*“I cannot find the foods that I have been trained on, so the training becomes useless. We don’t take them seriously because we don’t know what they are*…” **(***CG_011; Mother, 40)* *“The teachings were good but there was no money, we were required to give the baby milk, but you don’t have the money [for purchasing the prescribed food].” (CG_024; Mother, 33)*
High workload for women	Lack of time, too occupied with childcare & household activities	*“At times one has a schedule of jobs to do, like us who have to take care of small children. You want to go to the farm; at times you want to go to the market and that is why you may not be able to attend nutritional counselling” (CG_014; Mother, 37)* *“The training can be brought there, but you have work, like now people go to the farm…” (CG_030; Mother, 30)*
Sickness	Fatigue & sickness	*“The other thing that can hinder someone from going is sickness*,” *(CG_022; Grandmother, 68)*

### Preferences and recommendations for BCC with NHC

According to the caregivers and stakeholders, there are seven key features of a successful NHC for young children in Siaya County. Six of the features are shared by the caregivers and stakeholders, while one of the recommendations was mentioned by the stakeholders only.

Shared recommendations from caregivers and stakeholders:

1) Community-based setting: Both caregivers and stakeholders preferred a community setting, “ *I would want them (counsellors) to come home” (CG_008; Mother, 24)*,

*“I think for me, in the community works best, because you know, the people, whoever you are counselling is within their environment, they are relaxed…. if you’re in the confines of a health facility, they [participants] may not be that free to tell you, ‘We are not able to do this, we are not able to do the other’…” (SH_024; Nutritionist/Academia, 60).*

2) Male and female audience: Caregivers preferred that the mother is targeted for counselling and suggested that spouses could also be included “*The mother of the child, but both of you can be counselled but especially it should be the mother of the child.”, (CG_006; Mother, 28).* Likewise, stakeholders confirmed that they recognize the importance of male involvement in child feeding, hence preferred to target both parents for the counselling, *“We mainly prioritize the mothers but that doesn’t mean that we don’t focus on other household members because there is no point of giving information to a mother and they don’t have the control of the decisions that are made at the household level”, (SH_028; Program officer/International Organization, 38*).

3) Duration between 1 and 2 h: The views from both participant groups converged on an average of 60 to 120 min of the NHC session, ***“****Sitting down the way we are sitting should take around one hour”, (CG_ 012; Mother, 33)*, *“For the women, I think is that we need to take between one to two hours” ****(****SH_012; NGO, 45).* The setting may dictate the length, “*monthly group trainings, that would take about 60 minutes. But on the household visits that’s shorter, because you don’t want to take so much of their time, they’re working…” (SH_25,26,27; Program officers/International Organization; 27,28,33).*

4) Monthly frequency: Both participant groups thought 1 to 2 sessions a month would be sufficient, *“In a month, may be once (HH_012; Mother, 33), “…and the community health workers are supposed to visit every household on a monthly basis.” (SH_013; Director/GOK, 41).*

5) CHVs as counsellors: The caregivers preferred to receive counselling from CHVs. Caregivers liked and trusted the CHVs, “*Since she is the community health volunteer of this area, whatever she tells you is something you feel she has experienced because she also has her own children who are much older and even grandchildren, she* [the CHV] *can’t mislead you, (CG_019; Mother, 25).* Also, stakeholders preferred CHVs over facility-based health workers, “*the counsellors can be very few, visiting a very large community”, (SH_018; Program officer/NGO; 64).* At the same time, they recommend a good mentorship of the CHVs to equip them with the correct content, *“whoever is doing it has to be really well-trained and retrained because there is a lot of misinformation out here, on nutrition issues” (SH_011; Program officer/NGO, 40).*

6) Mix of individual and group counselling: There were divergent views from caregivers on whether to have individual or group counselling, *“…being visited at home individually” (CG_006; Mother, 28)* was preferred by some but not all caregivers, *“It’s good if it’s done in a group so that everyone benefits” (CG_008; Mother, 24).* This split was also evident in the stakeholders’ preferences. Some opted for individual approaches, *“nutrition counselling is much more personalized you cannot do a group counselling […] counselling is more personal, and it is tailor-made per each individual” (SH_007; Nutritionist/NGO, 34)*, while others strongly recommended the group sessions, “*I think this works a lot for most of our programs, is the group approach” (SH_012; Program officer/NGO; 34).* Some recommended a combined approach, *“well, for the group counselling, it was working easier at the community but for the individual counselling, one-on-one counselling it was much more at the facility” (SH_021; Nutritionist/ NGO, 33).*

Stakeholders’ recommendation:

Contextualized and evidence-based content: The stakeholders recommended using simplified but factual messages that address the local nutrition and health issues, *“proper information and information delivered correctly, I think those are two very important, the kind of information should be correct, and then, it should be delivered in a way it gets to people in the right language, in the right note” (SH_014; Nutritionist/ Academia, 39).*

## Discussion

### Summary of main findings

**Fig. 4:**
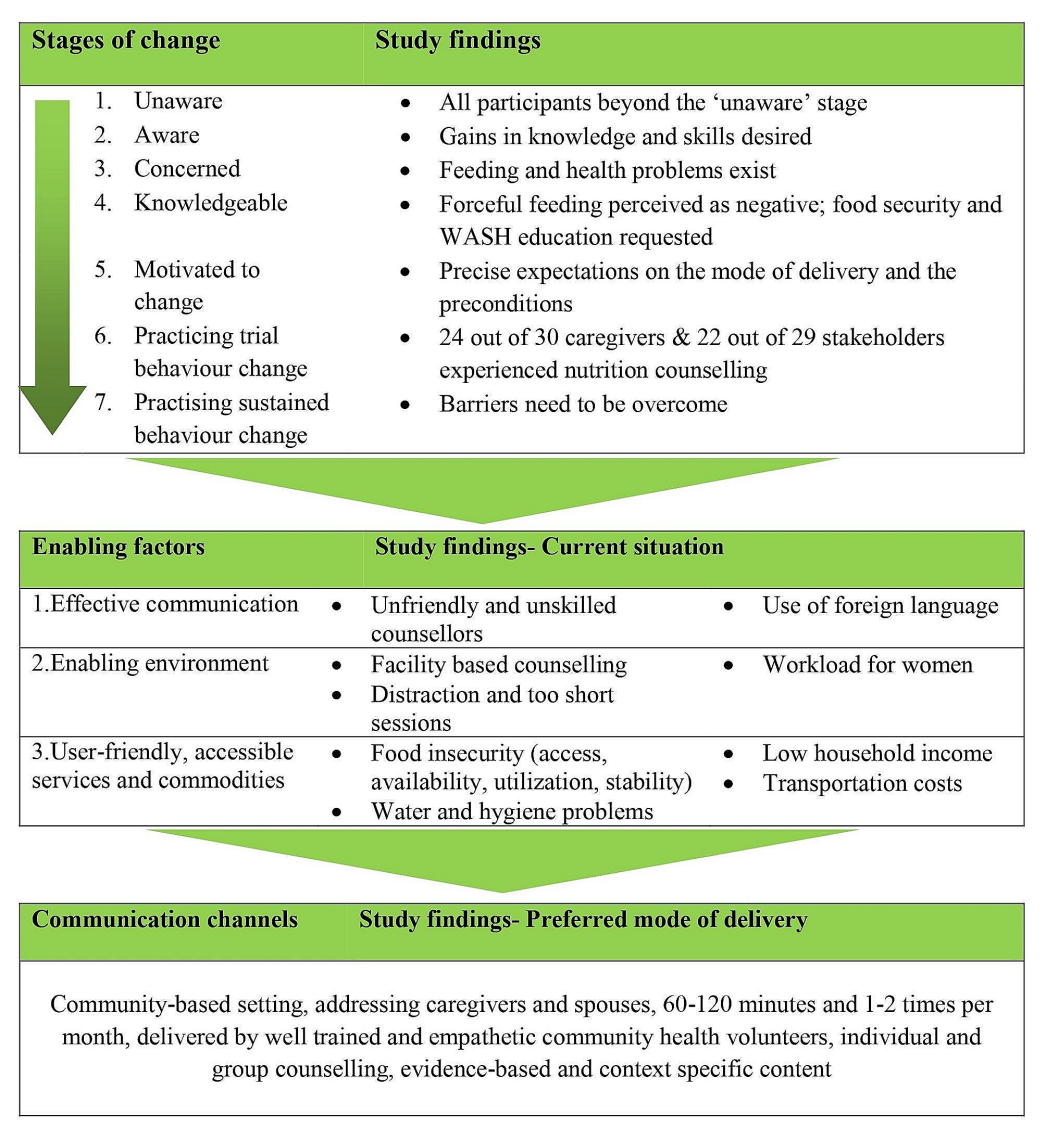
Summary of findings

This study explored caregivers’ and stakeholders’ experiences and preferences regarding nutrition and health counselling for young children in Siaya County, Kenya. As opposed to hospital-based counselling that caregivers had experienced before, the study participants (caregivers and stakeholders) preferred community-based services by well-trained CHVs. They highlighted the current barriers, including long queues and delays, long distances, high travel costs, the inapplicability of the counselling content, lack of spousal support, and a high domestic workload. Under the observed facilitating factors, such as trust in CHVs and services offered free of charge, both interview groups preferred that CHVs deliver the counselling, either to individuals or groups of caregivers and ideally, to both male and female caregivers (Fig. 4).

For the interpretation of our findings, we employ an integrated BCC framework, grounded in (1) behaviour change theories, such as the transtheoretical Stages of Change Model, (2) the expected and observed barriers and facilitators to deliver the BCC to the target community, and (3) relevant communication channels to successfully achieve the desired behaviour change [34]. BCC which uses nutrition and health counselling as a channel to communicate key messages on child nutrition has been applied in many settings across the globe and is associated with an increase in nutrition knowledge and improved feeding and health practices [5, 9, 35–38].

### Stages of change

The transtheoretical model for BCC outlines seven stages of change, namely being ‘unaware’ (pre-contemplation), ‘aware’ or ‘concerned’ or ‘knowledgeable’ (contemplation), ‘motivated to change’ (preparation), ‘practicing trial behaviour change’ (action), and ‘practicing sustained behaviour change’ (maintenance) [34]. The participants of this study are situated along this continuum. Most caregivers and all the stakeholders appear to be aware of the unfavourable nutritional situation among young children in Siaya County. They have already moved beyond the pre-contemplation stage. In fact, many of the participants were open to gaining more knowledge and skills on healthy feeding practices and hygiene measures for their children. Also, the stakeholders expressed their concerns about the nutrition and health situation of young children in Siaya County and emphasized the need for BCC strategies. In particular, a couple of caregivers and most stakeholders demonstrated profound knowledge about the pillars of food security, including availability, access, utilization, and stability [39], as well as water, sanitation, and hygiene (WASH) measures. Beyond this stage of being knowledgeable, a big share of the caregivers and stakeholders likely belong to the motivation stage, as they explicitly described the preconditions and preferred modes of delivery for nutrition and health counselling. Further, we observed that a considerable proportion of interviewees experienced or practiced trial behaviour. Yet, none of our participants seem to be situated at the stage of ‘practicing sustained behaviour’ because of the imbalances between barriers and enabling factors that were reported.

This situation is not unique to our study population. In nearby Kisumu, Reynolds and colleagues have conducted a Knowledge-attitude-practice (KAP) study among 20 mothers living in informal settlements [40]. They also saw that mothers generally know about feeding recommendations for young children, but caregivers cannot implement such practices due to similar barriers as observed in our study. Similarly, in rural Machakos, close to Nairobi, Uusimäki et al. confirmed that complementary feeding knowledge is good among mothers living in informal settlements [41]. In addition, in two neighbouring counties of Siaya, Homa Bay, and Migori, a recent qualitative study with 29 key informants revealed that caregivers are indeed knowledgeable and skilled enough to execute healthy child feeding practices. The authors, however, echo the findings of our study concerning social, financial, and cultural barriers that limit the adoption of recommended feeding behaviour [42]. To ensure that caregivers practice what they learnt in nutrition and health counselling, we also identified factors that contribute to the uptake of the desired practices.

### Enabling factors for behaviour change

Such factors for a successful BCC comprise: the communication of key messages must be effective, the environment in which messages are delivered ought to be conducive, and the services should be friendly and accessible. Regarding effective communication, the caregivers and stakeholders in this study highlighted that rigorous and repeated training of counsellors is key to effective nutrition and health counselling in Siaya County. This advice was given explicitly by stakeholders, as well as implicitly by caregivers. In their previous experiences with health professionals and CHVs, counsellors partly acted judgemental, unfriendly, unskilled, and unprofessional. Previous studies in rural and deprived settings in Kenya confirm the important role of CHVs in delivering messages about complementary feeding and general health. Yet, these studies have raised concerns about knowledge gaps and limitations in communication skills among CHVs, and thus, call for training them regularly on nutrition and health counselling [43–45]. Notably, this seems to be the situation in many low- and middle-income countries (LMICs). A recent umbrella review noted that CHVs constitute an immense potential in the formal health systems of LMICs. Still, CHVs are ineffective in handling complex situations, and therefore, require continuous training [46].

Concerning the enabling environment, both participant groups of the current study disliked the hospital as a setting of nutrition and health counselling for organizational, socio-cultural, and gender-related reasons. In fact, there is a rich body of evidence from nutrition and health counselling efforts in LMICs, including Bangladesh, Ethiopia, Uganda, and Malawi, that bi-weekly home visits offer more privacy, and thus, comfort, cognitive attention, and family support than on-demand counselling in public health facilities [47–52]. Regarding user-friendly and accessible services, in our study, the hospital-based setting was additionally criticized for the monetary and opportunity costs connected with the distance between caregivers’ homes and the health facilities. Clearly, when caregivers are highly motivated and ready to adopt healthy child feeding practices, the respective activities, services, and products should be accessible to them [34]. Systematically reviewed literature from home-based nutrition interventions in LMICs and *The Lancet* Series on Maternal and Child Nutrition 2 strongly argue for delivery strategies reaching the most vulnerable population groups at their homes [47, 53].

### Implications for nutrition and health counselling in Siaya County

Our study participants clearly stated their preferred mode of delivery for nutrition and health counselling to improve the nutritional status of young children in Siaya County, including the respective communication channels. Importantly, the caregivers’ and stakeholders’ ideas mirrored the current recommendations by the Government of Kenya on Baby-Friendly Community Initiatives (BFCI), which promotes maternal nutrition counselling to be delivered in a community setting [54]. In fact, our findings align with the recent efforts by the Kenya Ministry of Health to integrate the BFCI into the curriculum for CHVs [55] and thereby, delivering nutrition and health counselling at the community level. Taken together, best-practice examples, participant’s preferences and scientific evidence from other areas in Kenya and the Global South, suggest to thoroughly train the already well-trusted CHVs so that they can deliver household-based nutrition and health counselling. The sessions should last between one to two hours, take place once or twice per month, and address female and male caregivers [11, 48, 49, 51, 52, 56–58]. Consequently, BFCI should be routinely implemented everywhere in Kenya, particularly in rural areas where chronic child undernutrition hits every fifth child < 5 years of age.

### Strengths and limitations of the study

Our findings need to be interpreted with caution. While the conduct of most interviews in the Dholuo language was a strength during the interview process to facilitate the discussion of culture-specific and sensitive issues, the subsequent transcription into English might have led to a partial loss of the original meanings due to paraphrasing. Owing to the COVID-19 pandemic, we were unable to conduct interviews with stakeholders in-person. Rather, we used an online conference tool to facilitate data collection. This might have led to inconsistencies in data collection between the target groups, particularly referring to limitations in the record of non-verbal expressions. Still, the study team conducted debriefings as outlined above for both, the stakeholders’ and the caregivers’ interviews, to ameliorate this constraint. Another strength of the study was the ability to reduce subjectivity by peer coding. During data analysis, peer coding took place with the code books shared between the two coders to allow for the comprehensive identification of themes. Secondly, this study incorporated views from both caregivers and stakeholders, with many avenues for data triangulation and thus, validation of key themes. Thirdly, by adhering to the COREQ guidelines throughout the research process, we have enhanced the transferability of our findings [17, 59].

## Conclusion

This qualitative study with caregivers of young children and stakeholders in Kenya reveals that caregivers are highly motivated to change child feeding practices and healthcare behaviour towards improved food security and hygiene measures. Yet, they have challenges accessing counselling services in the current hospital setting and perceive this setting as inappropriate and inconducive. Caregivers and stakeholders prefer a change of the setting and the mode of delivery. This shows a disconnect between what is currently offered and what caregivers find helpful. At the same time, these observations corroborate current governmental efforts to train CHVs on delivering nutrition and health counselling at the community level. It has become evident that effective BCC needs to be tailored to the communication channels to this target population in rural Kenya. Precisely, counselling should be community-based, through trusted CHVs who deliver evidence-based content within less than 2 h every month; counselling should embrace male involvement and family support and allow for group and individual sessions.

### Electronic supplementary material

Below is the link to the electronic supplementary material.

Supplementary Material 1

Supplementary Material 2

Supplementary Material 3

## Data Availability

The data that support the findings of this study are available from the Steering Committee of the DFG-funded Research Unit “Climate change and health in sub-Saharan Africa”, coordinated by Ina Danquah: ina.danquah@uni-heidelberg.de but restrictions apply to the availability of these data, which were used under license for the current study, and so are not publicly available. Data are however available from the authors upon reasonable request and with permission of the Steering Committee of the DFG-funded Research Unit.
