# Supplementary material for: ‘Counselling is not just providing information’: perceptions of caregivers and stakeholders on the design of nutrition and health counselling interventions for families with young children in rural Kenya
Source: BMC Health Serv Res. 2024 May 7;24:597. doi: 10.1186/s12913-024-10872-w (PMC11077832; doi:10.1186/s12913-024-10872-w)
Supplement: Supplementary file 2 — Supplementary Material 2 [file 12913_2024_10872_MOESM2_ESM.pdf]

"Climate-sensitive nutrients, nutritional status and health in sub-Saharan Africa"

Interviewer: Complete these steps before starting the interview

|__| Introduce yourself and the DFG P2 project to the participant (mother of child <5)
|__| Ensure you are with the right participant in a place he/she can speak uninterrupted and freely
|__| Ensure the participant reads and understands the "Information Form" and the "Consent Form"
|__| Fill out and sign the "Consent Form" (2 copies, one for the interviewer & one for the participant)
|__| Fill out the "Participant Identification Form"
|__| Fill out the "Study Questionnaire"
|__| Turn on your audio recording device to start the interview

Interviewer: Introduce the structure of the planned interview. State that you will ask some general questions and that you then move to the experiences of the participant with home gardening and/or nutrition counseling.

Introduction

1. Please introduce yourself and your household.
2. Please tell me about your work and role in the community.

Nutrition Counseling

3. What do you know/have you heard about NC?
4. What makes it favorable/easy/encourages someone to seek out/receive NC?
5. What makes it hard/difficult/ stops someone from seeking out/receiving NC?
6. Has someone in your household ever received NC (now or in the past)?

	**YES – Participant/household member has experience with** NC?	**NO – Participant and household member has NO experience with** NC?
7.	What was the reason for the NC?	What would you want to know during a NC visit?
8.	What was the structure of the NC?	What would be the best structure for NC?
9.	What was the result of the NC?	What could be the result of the NC?
10.	What did you not like about the NC?	What would you not like in/during a NC session?

11. What indigenous/traditional/local vegetables are consumed in your household?
12. What are the preferred indigenous/traditional/local vegetables of your young children?
13. What is the best way to improve the nutrition of your young children?

Interviewer: Introduce the specifics of the DFG planned interventions to the participant

Acceptability

14. How do you feel about the intervention described?
15. How do you perceive the amount of work/effort required to participate in the intervention?
16. How does the intervention fit your beliefs/values/morals?
17. What aspects of the intervention have you understood?
18. What would you be giving up to participate in this intervention?

19. How well/poorly do you think the intervention will improve the nutrition of your children?
20. How well/poorly would you be able to apply nutrition counseling recommendations after the project ends?

Closing

21. What are your final thoughts on NC?
22. What are your last thoughts on our planned Nutrition Intervention project?

Interviewer: Thank the participants for their time and efforts
