# Supplementary material for: ‘Counselling is not just providing information’: perceptions of caregivers and stakeholders on the design of nutrition and health counselling interventions for families with young children in rural Kenya
Source: BMC Health Serv Res. 2024 May 7;24:597. doi: 10.1186/s12913-024-10872-w (PMC11077832; doi:10.1186/s12913-024-10872-w)
Supplement: Supplementary file 3 — Supplementary Material 3 [file 12913_2024_10872_MOESM3_ESM.pdf]

"Climate-sensitive nutrients, nutritional status and health in sub-Saharan Africa"

Interviewer: Complete these steps before starting the interview
|__| Introduce yourself and the DFG P2 project to the participant (mother of child <5)
|__| Ensure you are with the right participant in a place he/she can speak uninterrupted and freely
|__| Ensure the participant reads and understands the "Information Form" and the "Consent Form"
|__| Fill out and sign the "Consent Form" (2 copies, one for the interviewer & one for the participant)
|__| Fill out the "Participant Identification Form"
|__| Fill out the "Study Questionnaire"
|__| Turn on your audio recording device to start the interview

Interviewer: Introduce the structure of the planned interview. State that you will ask some general questions and that you then move to the experiences of the participant with home gardening and/or nutrition counseling.

Introduction

1. Can you please introduce yourself?
2. Can you please introduce your Ministry/Organisation(M/O)?
3. Can you tell me about your work/role in the M/O?

Nutrition Counseling

4. What is your professional experience with Nutrition Counseling?
5. What is your M/O experience with NC?
6. Does your M/O have projects related to NC (now or in the past)?

	YES – M/O has experience with NC	NO – M/O has NO experience with NC
7.	What was the reason for the NC project?	What would be the reason for starting a NC project?
8.	What was the structure of the NC sessions?	What would be the best structure for a NC session?
9.	What were the results of the NC project?	What could be the results of the NC project?
10.	What are/were the preferred indigenous/traditional vegetables of young children during the project?	What could be the preferred indigenous/traditional vegetable of young children?
11.	What was successful in the NC project?	What would make a NC project successful?
12.	What was not successful in the NC project?	What would make a NC project unsuccessful?

Closing

13. What are your last comments/recommendations for our planned Nutrition and health counseling project?

Interviewer: Thank the participant for their time and efforts!
